# Biomechanical analysis of operations for chronic ankle instability

**DOI:** 10.1186/1757-1146-7-S1-A126

**Published:** 2014-04-08

**Authors:** Jeseong Ryu, Jongsang Son, Youngkoo Lee, Kyungtai Lee, Youngho Kim

**Affiliations:** 1 Department of Biomedical Engineering, Yonsei University, Wonju, Gangwon, 220-710, Republic of Korea; 2 Department of Orthopedic Surgery, Soonchunhyang University, Bucheon, Gyeonggi, 420-767, Republic of Korea; 3 Foot and Ankle Clinic, KT Lee’s Orthopedic Hospital, Seoul, 135-820, Republic of Korea

## 

Ankle sprains are one of the most common sports injuries which are about 40% of total sports injuries and 20~40% of them would be progressed to chronic ankle instability (CAI) [1]. Rehabilitation and surgical therapy have been used to treat CAI, and the open modified Brostrm operation (MBO) is the gold standard surgical procedure. There are various evaluation methods in CAI treatments, such as the interview, visual analogue scale (VAS), and the measurement of range of motion (ROM) and the ankle torque. However, in reality, it is difficult to measure the maximum ROM and torque. Therefore, some studies measured ankle ROM and torque with cadaver specimens [2,3]. In this study, both open and arthroscopic MBO were performed on cadavers, and ankle torque and angle were measured during ankle inversion using the axial-torsion testing system. Ankle stiffness was calculated from measured data, and effects of both operations were compared quantitatively.

For this study, matched pairs of fresh-frozen human cadaver lower leg specimens were obtained from seven males and four females (average age 71.5 (range 58–98) years). Each specimen consisted of the distal half of a leg. The soft tissues were removed from the calcaneus and distal tibiofibular part, except for the ankle joint and ligament. The anterior talofibular ligament (ATFL) and the calcaneofibular ligament (CFL) were transected. The specimens for the arthroscopic and open MBO were chosen from the left and right legs alternately. Then, each specimen was fixed in the specially-designed jig that was mounted on the axial-torsion fatigue testing system (Instron 8874, Norwood, MA, USA). The test consisted of a single ramp from 0° to 70° by inverting the ankle at 5°/s, while measuring the angular position and resultant torque.

**Figure 1:**
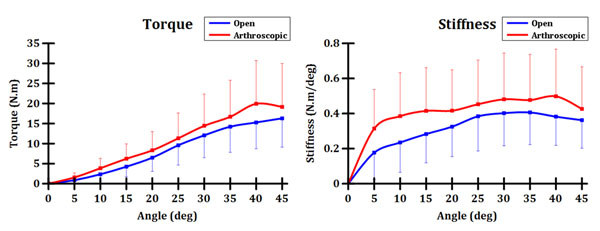
Ankle torque and stiffness during inversion test during open and arthroscopic MBO

There was no statistical difference in torque to failure between open and arthroscopic MBO. The maximum torque was 16.3±7.2 N•m at 45° for open MBO and 19.9±10.8 N•m at 40° for arthroscopic MBO (Figure 1). Ankle stiffness increased faster in arthroscopic MBO (0.31±0.22 N•m/deg) than in open MBO (0.18±0.15 N•m/deg) in initial inversion range (<5°), but no statistically significant differences were observed. The maximum stiffness was 0.41±0.18 N•m/deg at 35° in open MBO and 0.50±0.27 N•m/deg at 40° in arthroscopic MBO (Figure 1).

In this study, biomechanical analysis was performed for operations of CAI and there was no statistically significance in torque and stiffness. In comparison to open MBO, arthroscopic MBO is good alternative technique for CAI.
